# Superior nanopatterns *via* adjustable nanoimprint lithography on aluminum oxide in high-K thin films with ultraviolet curable polymer

**DOI:** 10.1039/d1ra08425a

**Published:** 2021-12-20

**Authors:** Jin Young Oh, Eun-Mi Kim, Gi-Seok Heo, Dong Hyun Kim, DongWook Lee, Hae-Chang Jeong, Dae-Shik Seo

**Affiliations:** IT Nano Electronic Device Laboratory, Department of Electrical and Electronic Engineering, Yonsei University 134 Shinchon-Dong, Seodaemun-gu Seoul 120-749 Republic of Korea dsseo@yonsei.ac.kr; National Center for Nanoprocess and Equipment, Korea Institute of Industrial Technology 6 Cheomdangwagi-ro 208beon-gil, Buk-gu Gwangju 500-480 South Korea; Electrical Engineering, Changwon National University 20 changwondaehak-ro, Unichang-gu Changwon Gyeonnam 51140 Korea jhchang@changwon.ac.kr

## Abstract

The present study substantiate that ultraviolet-nanoimprint lithography (UV-NIL) can be used to transfer a one-dimensional nano-pattern onto a high-k thin film of aluminum oxide mixed with a UV photocuring agent. Polydimethylsiloxane (PDMS) molds fabricated on silicon wafers were made using deep ultraviolet laser interference lithography in order to investigate one-dimension nanopatterns. These imprinted nano-patterns induce geometric deformations in the liquid crystal (LC), creating collective and elastic properties, which act as a guide for homogeneous alignment. The nanoimprint method can process a large area, so it can be processed much easier, faster, and more accurately than the conventional rubbing method. Moreover, the optical properties of the nano-imprinted aluminum oxide (AlO) thin-film are about 1.5p% superior to that of conventional commercialized cells, so it has a high effect on the luminance and color gamut of the display. After pattern imprinting, atomic force microscope (AFM) was performed to confirm the result. We can compared the cycle of AlO mixed with UV photocuring agent PDMS pattern cycle, the period is 776 and 750 nm, the width is 468 and 450 nm, the spacing is 292 and 300 nm, and the height is 40 and 30 nm. The nano-imprinted film appears to replicate the width, amplitude, and spacing of the PDMS template. In addition, X-ray photoelectron spectroscopy was performed to determine the chemical properties of the thin film and it was confirmed that UV irradiation induces oxidation, thus increases the intensity significantly. The binding energies of Al 2p and C–O spectra were situated at 74.27 ± 0.5 eV and 531.78 ± 0.5 eV, respectively.

## Abstract 2

The present study substantiate that ultraviolet-nanoimprint lithography (UV-NIL) can be used to transfer a one-dimensional nano-pattern onto a high-k thin film of aluminum oxide mixed with a UV photocuring agent.

## Graphical abstract

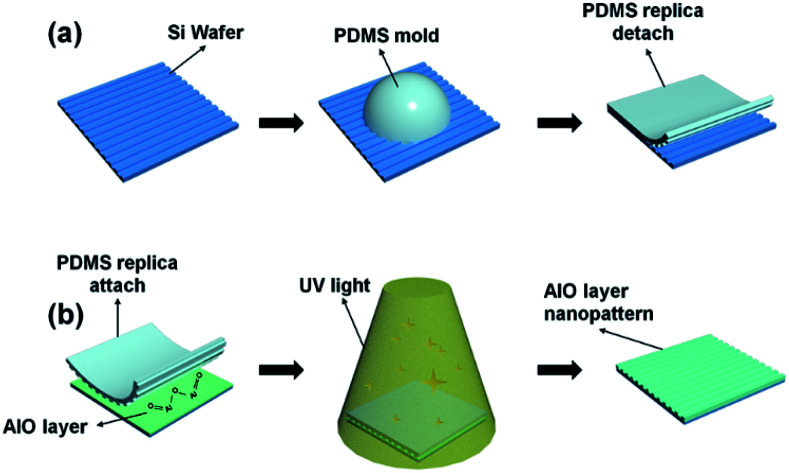

## 1. Introduction

The display industry is becoming essential in various fields our lives. Such as smartphone, computers, HUDs, AR/VR, *etc.* based on various chemical, optical, and electrical characteristics. Liquid crystal displays (LCDs) are still in high demand the world and a lot of progress has been made by applying light-emitting diodes and quantum dots. Moreover, numerous researchers have investigated the properties of organic and inorganic films.^1–3^ Liquid crystals (LCs) were studied for the alignment of various organic and inorganic this films to achieve advanced high resolution, thin thickness, and compatibility.^2^ In order to use the inorganic films as the LC alignment layer, an alignment methods similar to the rubbing process commonly used for the polyimide (PI) layer is required.^4–7^ This common rubbing method has several advantages, but depending on the nature of the contact alignment method, this method also has serious drawbacks in terms of debris generation, and localized defects.^8^ Many researchers have tried to overcome the shortcomings of various non-contact methods by developing methods such as photo alignment,^9,10^ oblique evaporation,^11,12^ ion-beam irradiation, ultraviolet (UV) irradiation, and nanoimprint lithography (NIL) using organic or inorganic materials.^13–16^

The UV NIL method, which is widely used in the TFT field, has advantages such as high throughput, low cost-effectiveness, large process, high thermal endurance, and low temperature processing. Therefore, using UV NIL method, we created a one-dimensional nano-pattern on an alignment layer for an LC cell. As a result, UV NIL can be used in various devices and applications such as photonics and light-emitting diodes in photovoltaic cells as well as for, tissue engineering in genomics and proteomics. In particular, UV NIL can be used in the manufacturing of flexible plastic electronic devices in a roll-to-roll process. More importantly, these advantages enable, the nanostructures of the LCs to uniformly align in the same direction.

The use of inorganic thin films for LC alignment is a famous concept in LCD technology. A variety of optically transparent and insulating films have been developed, including diamond likes carbon,^17^ SiN_*x*_,^18^ Ta_2_O_5_,^19^ TiO_2_,^20^ MoO_3_,^21^ and Al_2_O_3_ with or without nitrogen doping. The use of Al_2_O_3_ (ref. 22) in recent semiconductor thin-film transistors showed no hysteresis and no *V*_T_ shift, aluminum oxide are high-k materials and has suitable electrical properties for it to be used as a dielectric. In this study, we used high-k films as LC alignment layers *via* NIL with UV irradiation. Excellent LC alignment characteristics were achieved by UV beam evaporated amorphous Al_2_O_3_ films. Moreover, the LC alignment characteristics are function of the UV irradiating time. Various surface changes, which have a great influence on the excellent LC arrangement, according to the UV irradiation time were observed, by AFM and XPS. Furthermore, this was confirmed by the morphology and chemical changes of the thin film surface that enable the homogeneous alignment of the LC for the topographic grating of the one-dimensional nano-pattern served as the director care of LC alignment. To confirm this LC alignment, we used polarized optical microscope investigations and crystal rotation measurements.

## 2. Experiment

### 2.1 Fabrication of polydimethylsiloxane (PDMS) mold and of AlO solution

**Fig. 1:**
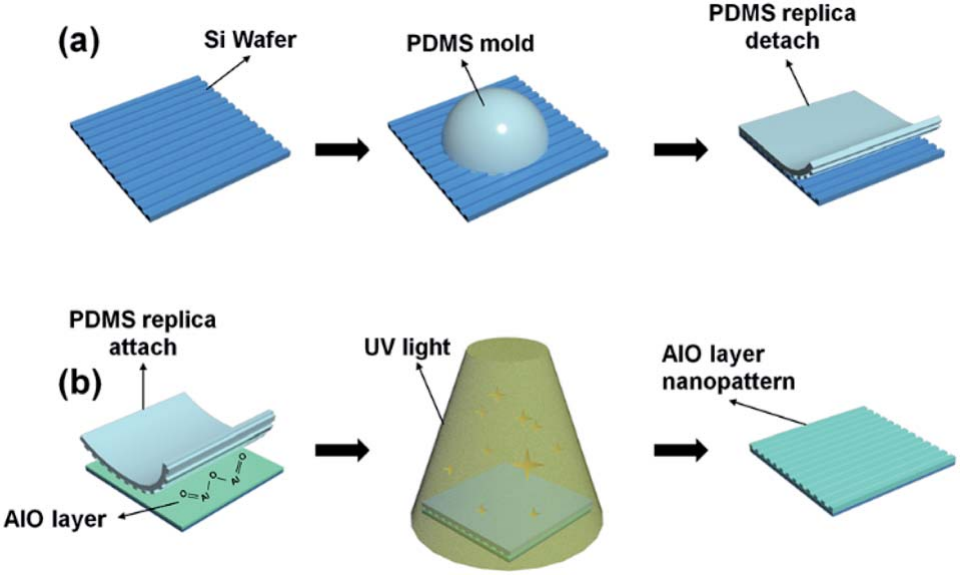
Schematics of (a) fabrication process of PDMS template on silicon wafer and (b) transfer of the nano pattern to aluminium oxide mixed with UV photocuring agent layers.

As shown in Fig. 1(a), PDMS (Sylgard-184, Dow corning) was prepared by mixing the elastomer base and curing agent in a proportion of 10 : 1 on a periodic nano-structure Si master. The Si master was fabricated using deep ultraviolet laser interference lithography. Furthermore, the mixture was placed in a vacuum box to remove air bubbles for 1 h and to harden the mold by heating at 80 °C and 2 h. Secondly, using Al(No_3_)·9H_2_O (Sigma Aldrich Korea Co.), an 0.1 mol L^−1^ AlO solution was prepared by mixing the aluminum nitrate nanohydrate ≥98% in 2-methoxyethanol (2ME). Then, the solution was stirred at 80 °C for 2 h using a hotplate magnetic stirrer and subsequently aged at room temperature for 1 day. The solution was mixed with a UV photocuring agent at a volume ratio of 10 : 1.

### 2.2 Shift a pattern from PDMS template to UV curable solution

**Fig. 2:**
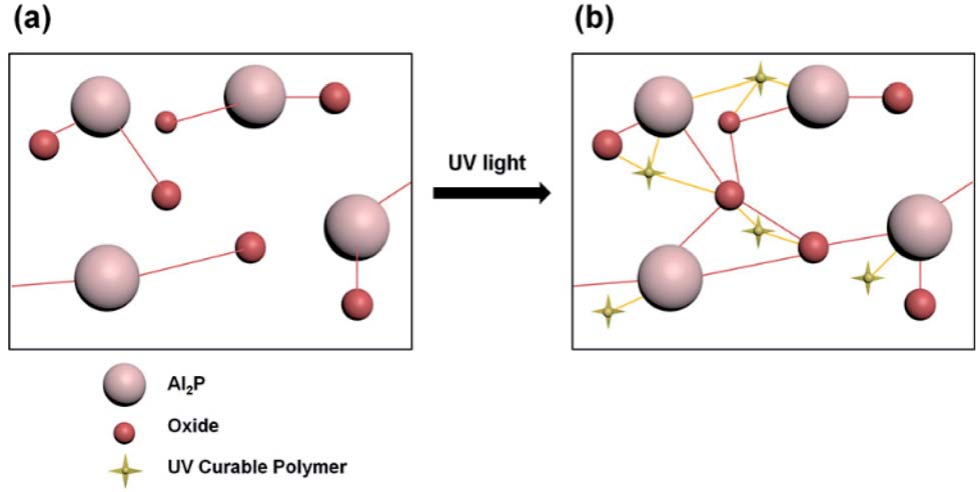
Reconstitution of molecules bonds by adding UV-curable polymers with UV irradiation (a) before, (b) after.

The UV photocuring agent (Irgacure 2959; Sigma-Aldrich, Ciba) contains three different components. First, dipentaerythritol hexaacrylate (DPEHA), a hexa-functional monomer used to produce a transparent hard coating to prevent static electricity. The second is tripropyleneglycol diacrylate (TPGDA), a trifunctional monomer used to increase the gelation stability at elevated temperatures with low viscosity, high *T*_g_, and fast cure speed. Last is 2-hydroxyethyl acrylate (HEA), a hydroxy-functional acrylic monomer usable in a variety of ways to produce resins that are useful in high-performance coating applications. Therefore, UV photocuring agent shown absolute reaction each molecule as shown in Fig. 2(a) and (b).

For low film thickness, the mixture solution was spin-coated onto a clean glass substrate at 3000 rpm for 30 s. Then, PDMS template imprints were coated onto the substrate with UV light exposure. The UV exposure was with a 200 W XeHg lamp at 365 nm with a transmission of 15% and an exposure time of 1, 4, and 7 min. The solution solidified with UV exposure and a shift PDMS stamp pattern was formed on the thin film. The film was then peeled away as shown in Fig. 1(b).

### 2.3 One dimensional nano pattern

**Fig. 3:**
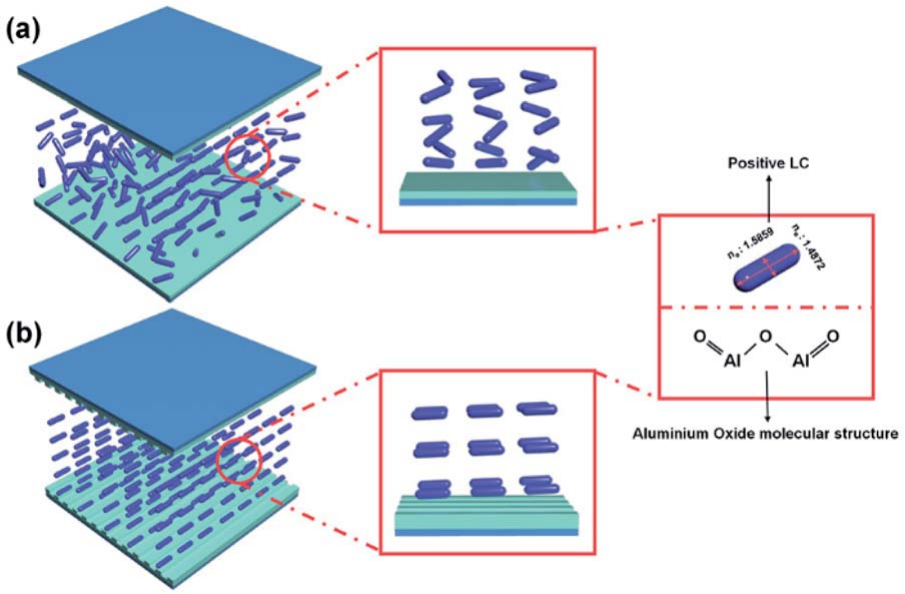
Alteration in positive LCs alignment according to nanoimprinting patterns (Δ*ε* = 8.2, *n*_e_ = 1.5859, *n*_o_ = 1.4872). (a) LCs no alignment on flat aluminium oxide film and (b) LCs alignment over nanopatterns composed of aluminium oxide.

As shown in Fig. 3(a) and (b), LC cells were fabricated in antiparallel with a cell gap of 60 μm to observe the polarized optical microscopy (POM; BXP 51, Olympus) and pretilt angles (TBA 107; Autronic) of the LC nematic phase using the crystal rotation method for alignment conditions. Positive LCs (Δ*ε* = 8.2, *n*_e_ = 1.5859, *n*_o_ = 1.4872) were purchased from Merck Corp. and injected into combined cells *via* capillary injection.

### 2.4 Measurement of the properties of thin-film nanoimprint patterns

To confirm the alignment properties, atomic force microscopy (AFM; Xe-100, Park system) was used to investigate the surface morphologies of the nano-patterns on the PDMS mold and thin film. The AFM measurements were recorded in non-contact mode (scan rate = 0.5 Hz, optical transmittance = 250–800 nm) at room temperature using an ultraviolet-visible near-infrared scanning spectrophotometer (UV-3101PC, Shimadzu). The chemical compositions of the AlO mixed with UV photocuring agent were investigated by X-ray photoelectron spectroscopy (XPS; ES-CALAB 220i-XL, VG Scientific). The XPS measurement principle uses a monochromatic X-ray source where X-ray collide with parallel crystal planes at an *θ* angle and reflect at same angle as well. Furthermore, the Bragg equation (*nλ* = 2*d* sin *θ*) and X-ray monochromators, in combination with K-alpha radiation, are also used. Using these principles, we can confirm the binding energies of the Al, C, O atoms of the main chemical components.

## 3. Result and discussion

### 3.1 Measurement of transmittance of UV-vis graph

Transparency (V-730 UV/VIS Spectrophotometer, JASCO) is an essential factor for the application of display devices. The fundamentals of the spectrophotometer are that when an atom or molecule receives light energy from the outside, electrons in the atom or molecular orbital absorb the light and cause a transition. At this time, only light with the same energy as the amount of energy required for transition is absorbed. Light energy is proportional to the wavelength as shown in the equation below, it absorbs visible light of a different wavelength from UV light according to the energy required for transition. *E* is the light energy, *h* is Frank's constant and *v* is frequency as the following eqn (1).

1 *E* = *h* × *vE*

2
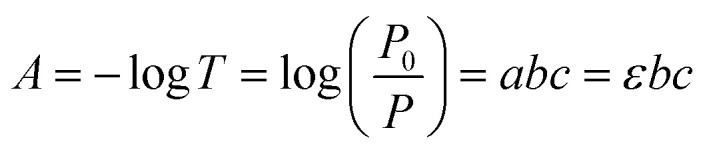


**Fig. 4:**
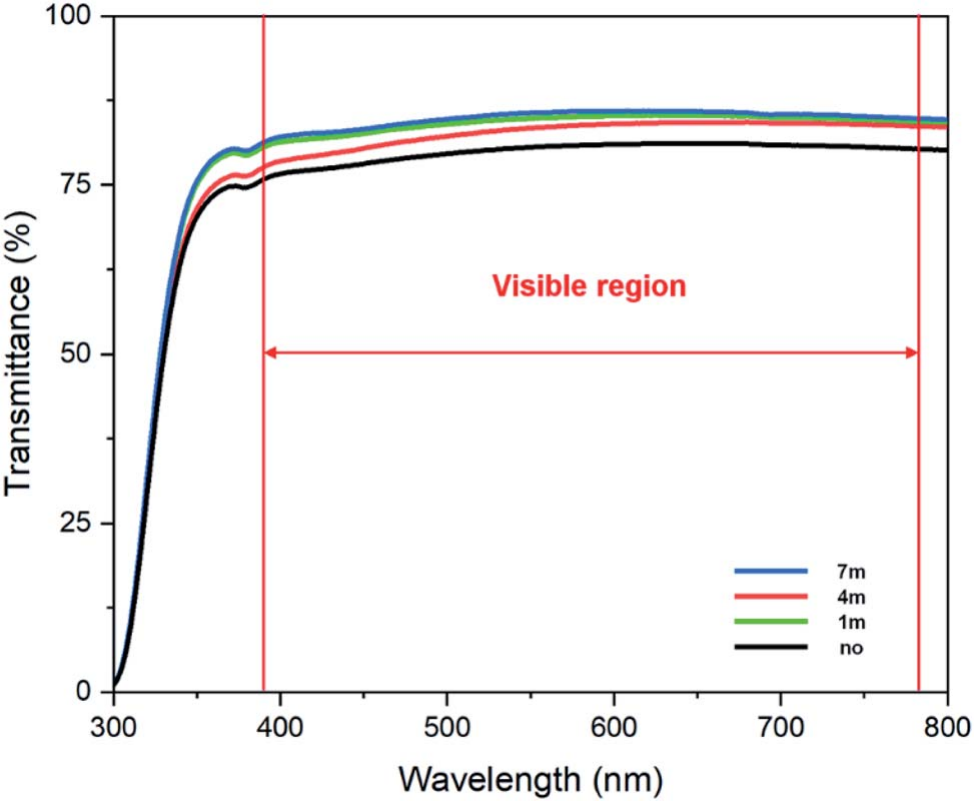
Transmittance of UV-vis graph of UV irradiated to 1 m, 4 m, 7 m, and non-patterned on AlO with UV photocuring agent thin film.

Furthermore, Beer–Lambert law eqn (2) is an expression of the linear relationship between the absorption concentration of electromagnetic waves and the absorption values. Where *P* and *P*_0_ are intensity of radiation of light energy per 1 cm^2^ area of the detector per second, *A* is absorbance, *T* is transmittance, *b* is passage of radiation, *a* is absorption coefficient and *ε* is molar extinction coefficient. As shown in Fig. 4, in the visible light region (380–780 nm), the average optical transmittances of the single deposited UV irradiated nano-patterned layer are 79.98%, 84.29%, 82.79%, 84.92% for non-irradiated, 1 m, 4 m, 7 m, respectively. For comparison, the transmittance of conventional PI coated glass has an average transmittance of 83.23%. As expected, the optical transparency properties of invested nano-pattern layers typically increase as photons from visible light are trapped by the periodic nanostructure according to the waveguide mode theory.^23^ Furthermore, the transmittance increased when AlO is mixed with the UV photocuring agent layer. As a result, a 1.5p% increase was observed for the sample irradiated for 7 min, unlike the non-irradiated substrate which decreased by about 3.3p% compared to the PI sample.

### 3.2 POM and pretilt measurement and analysis for LCs alignment

**Fig. 5:**
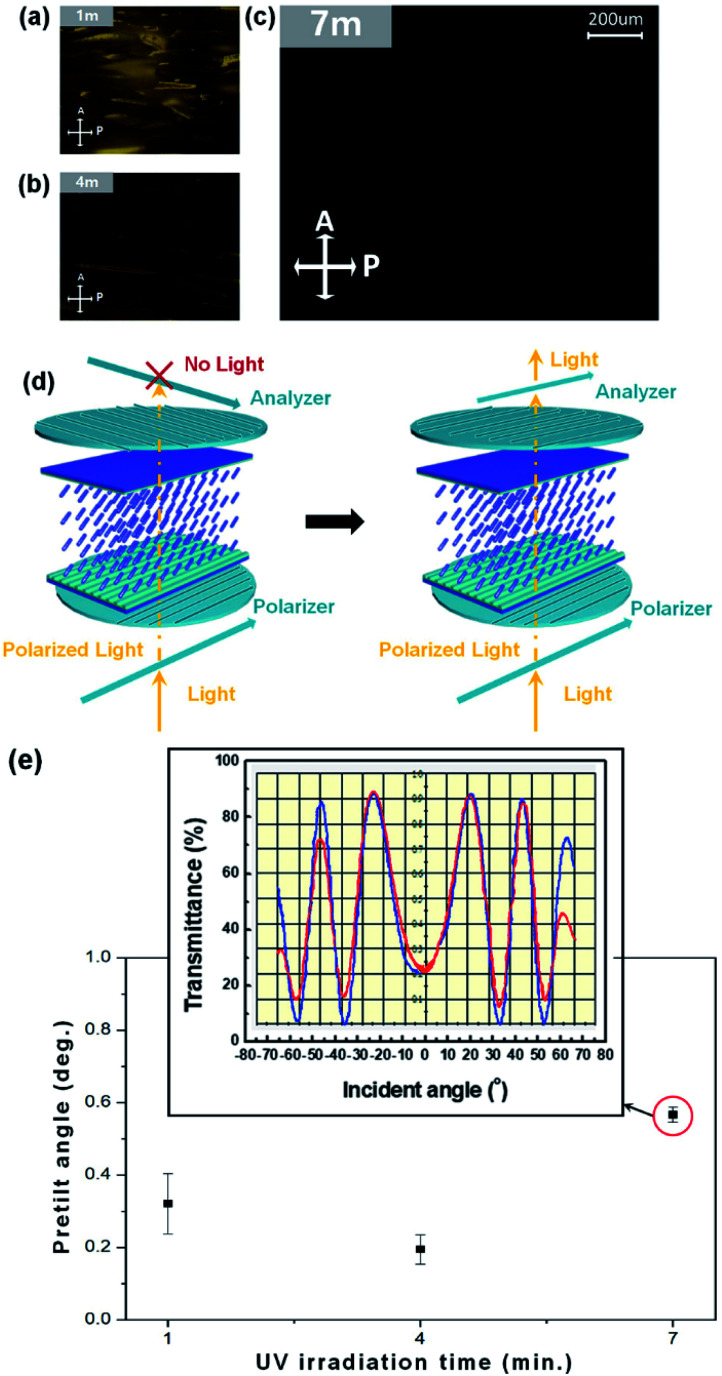
POM images of one dimension nano patterned on AlO mixed UV photocuring agent thin film using general glass substrates to twisted nematic (TN) liquid crystal (LC) cell *via* 60 μm gap with positive LCs (Δ*ε* = 802, *n*_e_ = 1.5859, *n*_o_ = 1.4872) follow UV irradiated (a) 1 m, (b) 4 m, (c) 7 m. (d) Principle of polarized optical microscopy. Transmittance curve of one dimension nano patterned on AlO mixed UV photocuring polymer this film using general glass substrates to twisted nematic (TN) liquid crystals cell of 60 μm with latitude rotation of the angle of incidence due to crystal rotation to measure the (e) pretilt angle which UV irradiated 1 m, 4 m, and 7 m.

LC alignment on the AlO mixed with UV photocuring agent layers is comparable to the UV irradiated time in an anti-parallel LC cell *via* POM. A micrograph of an antiparallel LC cell composed of a nano patterned AlO film is shown in Fig. 5. Initially, dark POM images were measured by crossed polarizers, where random alignment LC was observed in the solution layer of irradiated UV after 1 min in Fig. 5(a) and after 4 min in Fig. 5(b). On the other hand, stably aligned LC molecules in one direction after UV irradiation for 7 min are shown in Fig. 5(c). Hence, we confirmed the existence of homogeneously aligned LC on the nano-patterned UV cured AlO film. When the nanopattern is parallel to one axis of the cross polarizer, light propagates in a straight line through the LC molecules, arranged in parallel, and is blocked by the cross polarizer on the upper substrate. Evenly aligning the LC and rotating the cell 45° maximizes the transmittance of light passing through the cross polarizer. Therefore, we can confirm that the LC cell rotated under the cross polarizer maintains a uniform alignment. The direction of the nano-pattern was between the polarizing plates intersecting at an angle of 45° to the direction of the polarizing plate.

Circular polarization was observed due to the birefringence properties of the LC. Therefore, the light passing through the polarizing plate and the diagonal LC to the polarizing plate yielded the white POM image. Hence, we confirmed the existence of homogeneously aligned LCs on the nano-pattern. Fig. 5(d) shows a graph of the experimental curve (blue), which was extracted based on well-aligned LCs using birefringence information and simulated pre-tilt angle curves (red). The experimental curve coincides with the simulated curve, indicating that the LC molecules are well aligned along the same direction and that the pre-tilt angle was calculated with high confidence. As can be seen from the graph, the most uniform pre-tilt value can be seen during UV irradiation of 7 m, which confirms that the LCs are well arranged. Eventually, an average pre-tilt angle of 0.525998° at 7 m, consistent with the previously mentioned POM image analysis, was obtained for the LC of the nano patterned AlO mixed with UV photocuring agent film.

### 3.3 Determine of pattern transferred *via* analysis of AFM

**Fig. 6:**
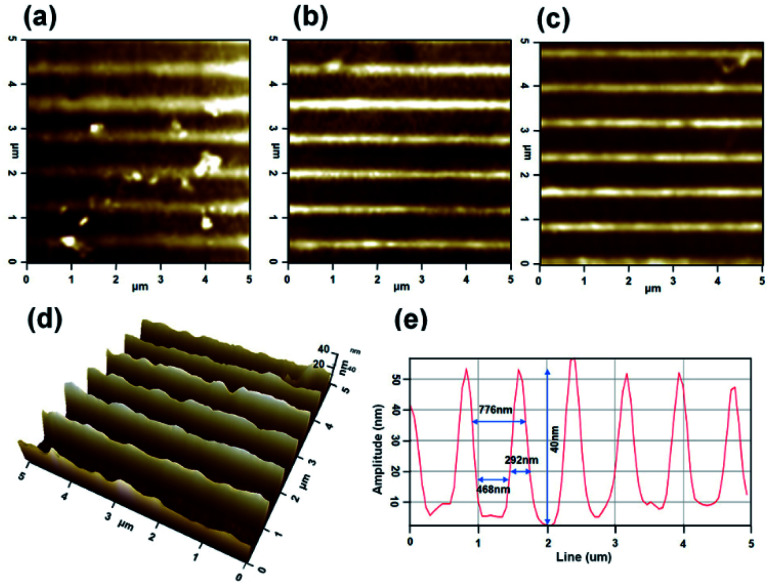
The AFM images in three dimensional of patterned transferred on AlO mixed with UV photocuring agent thin films to (a) UV irradiation of 1 m, (b) 4 m, (c) 7 m, and (d) 3D image of 7 m. (e) Line profiles of AlO thin film *via* UV irradiation of 7 m.

AFM images of the AlO mixed with UV photocuring agent are presented in Fig. 6. The figure shows the three-dimensional morphology and the cross-section of the surface of the PDMS template, before imprint and after pattern transfer. It can be seen that the PDMS template imprinted as a liquid state thin-film containing solution filling the dents of the nano-pattern. For the non-imprinted PDMS template, we can see the shape of a flat thin film. However, after imprinting the PDMS template with UV exposure, the AlO mixed with UV photocuring agent solidified and the pattern on the PDMS template was transferred onto thin film, creating a one-dimensional linear pattern. Based on these observations, a linear nano-pattern could be used to direct the alignment of LC molecules. Two things should be noted: first, the PDMS pattern had an average line pattern period of 750 nm, a width of 450 nm, a spacing of 300 nm, and a height of 30 nm. The pattern of AlO mixed with UV photocuring agent had an average line pattern period of 776 nm, a width of 468 nm, a spacing of 292 nm, and a height of 40 nm after 7 m of UV irradiated, as shown in Fig. 6(c). After the pattern imprinted, an inverse replica of the PDMS template with a similar width, spacing, and line period was obtained. Likewise, similar results were obtained after UV irradiation for 1 m and 4 m as shown in Fig. 6(a) and (b), respectively. However, it was confirmed that the height was approximately 23 nm and 33 nm for 1 m and 4 m, respectively, but this was still lower than the height of 7 m. Second, a height difference was observed between the patterns of the PDMS templates after 30 nm and the AlO mixed with UV photocuring agent after 40 nm. This is a result of the combination of strain relaxation and recovery of the cured polymer, which is one of the demolding issues reported for UV-cured polymers.^24^ During the curing process, the poly-function polymer facilitates the formation of a three-dimensional network.^25^ This enables free spacing on the polymer, which may result in volume expansion.^26^ Moreover, as the volume expands, an investigation into the aligned patterns shows the same morphologies as the PDMS templates.

### 3.4 XPS analysis of AlO spectra change according to UV irradiation

**Fig. 7:**
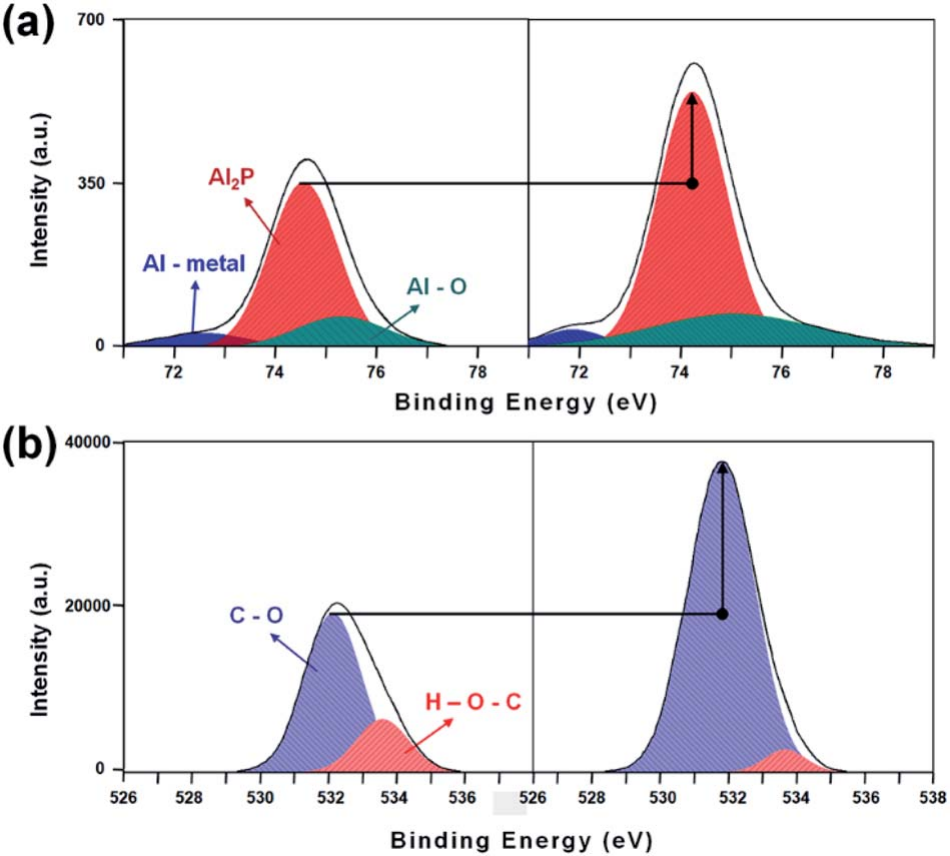
XPS spectra for (a) Al 2p, and (b) O 1s of the Al_2_O_3_ surface between UV irradiation or non.

We demonstrated the results of an AlO solution with a UV curable polymer pattern applied the LC alignment cells. Aluminum nitrate nanohydrate, Al(No_3_)·9H_2_O, oxidizes upon irradiation with UV. In the XPS analysis presented, Al 2p, C, and O elements were discovered in the XPS spectra. The binding energies of Al 2p and C–O spectra were situated at 74.27 ± 0.5 eV and 531.78 ± 0.5 eV, respectively. As shown in Fig. 7(a) and (b), the binding energies exhibit no shift due to UV irradiation, however, large changes in the intensities of the Al 2p and O 1s peaks were observed. Typically, a UV curable polymer is mainly composed of carbon and oxygen bonds, mostly as carboxyl groups. UV irradiation causes oxidation of the aligned surfaces, where the chemical states of the atomic deposited Al_2_O_3_ films show different characteristics *via* UV irradiation, which influences various properties during the crosslinking reaction.

## 4. Conclusions

In summary, we investigated a hyper aligned layer candidate based on a UV-NIL method for commercial LCDs fabrication. We experimentally produced a homogeneously aligned LC *via* nano imprinting patterned AlO mixed with UV photocuring agent. To investigate the nano-pattern, a PDMS mold was fabricated on a Si wafer made in one dimension *via* deep UV-NIL. Then, a thin film was prepared using a solution of a UV curing agent mixed with AlO, covered with a PDMS template, and imprinted with a nano-pattern through UV irradiation. We confirmed that the hydrophilic UV-cured polymer surface enabled the homogeneous alignment of the liquid crystal while the topographic grating of the one-dimensional nano-pattern served as the director for uni-directional LC alignment. This was confirmed by contact angle measurements, polarized optical microscope investigations, and crystal rotation measurements. The nanoimprint method can process a large area, so it can be processed much easier, faster, and more accurately than the conventional rubbing method. As follows, the one-dimensional nanopattern induced geometric deformations in the LC to generate collective and elastic properties, while acting as a guide to align the LC in the same direction. Finally, good transparency was confirmed with AFM and XPS analysis. Moreover, changes in the chemical properties through UV irradiation brought about the greatest change, and the LC alignment was possible due to these new properties. Furthermore, a substrate with a low pretilt angle could be used to form anisotropic nanopatterns. Accordingly, the nano patterned AlO thin films, mixed with UV photocuring agent, show great potential for flexible, rollable, and other superior LC devices.

## Author contributions

Jin Young Oh: UV NIL process, LC cell production, paper writing, Eun-Mi Kim: AFM measurement. Gi-Seok Heo: AFM measurement. Dong Hyun Kim: POM measurement. DongWook Lee: POM measurement. Hae-Chang Jeong: XPS analysis, project manager. Dae-Shik Seo: AFM analysis, supervision.

## Conflicts of interest

There are no conflicts to declare.

## Supplementary Material
